# Network architecture associated with the highly specialized hindlimb of frogs

**DOI:** 10.1371/journal.pone.0177819

**Published:** 2017-05-17

**Authors:** Daniel Andrés Dos Santos, Jéssica Fratani, María Laura Ponssa, Virginia Abdala

**Affiliations:** 1 Instituto de Biodiversidad Neotropical, CONICET-UNT, Tucumán, Argentina; 2 Unidad Ejecutora Lillo, CONICET-FML, San Miguel de Tucumán, Tucumán, Argentina; 3 Cátedra de Biología General, Facultad de Ciencias Naturales e IML, Universidad Nacional de Tucumán, San Miguel de Tucumán, Tucumán, Argentina; Universidad Rey Juan Carlos, SPAIN

## Abstract

Network analyses have been increasingly used in the context of comparative vertebrate morphology. The structural units of the vertebrate body are treated as discrete elements (nodes) of a network, whose interactions at their physical contacts (links) determine the phenotypic modules. Here, we use the network approach to study the organization of the locomotor system underlying the hindlimb of frogs. Nodes correspond to fibrous knots, skeletal and muscular units. Edges encode the ligamentous and monoaxial tendinous connections in addition to joints. Our main hypotheses are that: (1) the higher centrality scores (measured as betweenness) are recorded for fibrous elements belonging to the connective system, (2) the organization of the musculoskeletal network belongs to a non-trivial modular architecture and (3) the modules in the hindlimb reflect functional and/or developmental constraints. We confirm all our hypotheses except for the first one, since bones overpass the fibrous knots in terms of centrality. Functionally, there is a correlation between the proximal-to-distal succession of modules and the progressive recruitment of elements involved with the motion of joints during jumping. From a developmental perspective, there is a correspondence between the order of the betweenness scores and the ontogenetic chronology of hindlimbs in tetrapods. Modular architecture seems to be a successful organization, providing of the building blocks on which evolution forges the many different functional specializations that organisms exploit.

## Introduction

Network analyses have been increasingly used in the context of comparative vertebrate morphology due to their undoubtedly fresh perspective regarding issues such as anatomical topology, morphogenetic integration, and modularity [1,2]. Addressing anatomical systems as networks and exploring their patterns of connections can lead to useful insights. For instance, one of the most interesting properties of networks is intermediacy in information flow, and the betweenness score as a measure of centrality can be used for detecting the bridging role a given node can achieve in the flow of information through the network [3]. The development of a novel methodology for anatomical studies, such as anatomical network analysis (AnNA, [1]) has allowed treating the skeletal units of the vertebrate body as elements of a network (nodes), whose interactions at their physical contacts (links) determine the phenotypic modules, with an implicit correspondence between topology and anatomical organization.

Individuals are composed of several parts that are more or less different of each other in terms of function, anatomical structure, and embryological origins. This organization in subunits implies a modular organization [4–13]. Complex systems often evolve in a modular fashion, i.e. with traits integrated into groups (modules), which can then change in a coordinated manner and independently of other modules [13]. In the framework of evolutionary biology, a morphological module is a complex of phenotypical characters that meet three criteria: 1) they serve together to a primary function, 2) they are integrated by strong pleiotropic effects of genetic variation, and 3) they are relatively independent from other units [14–16]. Recently, several studies assessing morphological evolution have focused on modularity, including studies of the evolution of insect wings [17–20], rodent mandibles [21–29], and skulls of lizards [30], birds [31] and various mammals, including humans [2,32–42].

The simplest definition of a morphological module refers to a semi-independent set of body elements with more numerous and stronger interactions among themselves than with outer elements [16]. In the context of network analysis, modules are sets of densely connected nodes more strongly linked to each other than to the rest of the network. In addition to having the same name, the deep similarity between the definitions for both concepts (i.e. morphological and structural modules) is remarkable. Thus, the quantitative procedures and algorithms developed for community detection in networks, and built around the notion of modularity [3], have attracted the attention of morphologists to shed light about module issues in comparative biology [16]. Morphological modularity remains poorly studied both in basal lineages of tetrapods as well as in key anatomical systems, but the motivation to fill these gaps of information increases greatly with these new opportunities of research. For instance, the modularity of the tetrapod limbs is crucial to understand changes associated to different locomotion modes. Certainly, a network approach could lead to new insights about the integration of the musculoskeletal system in limbs, and an example in such direction is a recently published study on humans [43].

Among vertebrates, network modularity studies might be especially important in anurans. All anurans can jump, an activity that can be described as the paired extension of hindlimbs propelling the animal off the ground [44]. The specialized morphology associated to jumping in frogs includes a shortened trunk and tail, elongated ilia, and elongated hindlimbs [45–47]. These morphological traits were already present in the earliest fossils such as *Prosalirus* [47,48]. Even though the biomechanics of jumping in anurans is reasonably simple [49], several morphological features potentially determine the ability to jump of a given animal, and they are generally dependent of each other. The exclusive combinations of structural and functional properties of each specimen will determine its locomotion performance, which may originate from their individual elements in non-obvious ways [50]. Delimiting modules and analyzing their correspondence with anatomical features is a big contribution to the understanding of the development and evolution of morphological structures [51]. The identification of modules can also contribute to the understanding of the ways by which the anatomical parts of the body of an animal evolve into very different forms, but still fit together and function properly [52–56].

Here, we use a graph-theoretic approach to model the hindlimb of frogs as an anatomical network in which the fibrous knots, the skeletal and muscular units are represented as nodes, while their physical contacts are represented as edges. The resultant network model is then used to further identify patterns of organization. Our main hypotheses sustain that (1) the higher betweenness values are recorded for fibrous elements belonging to the connective system, (2) the musculoskeletal network exhibits a modular organization or non-trivial architecture, (3) the modules in the hindlimb reflect functional and/or developmental constraints, and these restrictions are not necessarily overlapped.

## Material and methods

### Anatomical data and network elements

In this paper, we studied the musculoskeletal and tendinous anatomy of the pelvic girdle and hindlimb of adult specimens of *Leptodactylus latinasus*. Specimens are housed in the herpetological collection of Fundación Miguel Lillo (FML). The list of examined specimens includes material from Argentina: 1) Jujuy, Aguas Blancas: FML 29482, 2) Salta, La Unión: FML 29480–81, 3) Salta, Orán: FML 29478–79, and 4) Tucumán, Lules, FincaNougués: FML 2983–84. Specimens were dissected and examined with a Zeiss Discovery. V8 Stereomicroscope. Anatomical nomenclature follows Diogo & Abdala [57], Diogo & Ziermann [58], and Diogo & Molnar [59]. Since some of the names used in [57–59] do not correspond to those most commonly used for anurans in the literature [60,61], we provide a list of equivalent nomenclatural terms (Table 1).

**Table 1:** Pelvic and limb muscles of adults of *Leptodactylus latinasus*, following the nomenclature of Diogo & Molnar [59] and synonyms commonly used in anuran literature.

Modern nomenclature [59]	Preterite nomenclature [60,61]
-	Coccygeosacralis
-	Coccygeoiliacus
Iliofemoralis	Iliofemoralis
Tenuissimus	Iliofibularis
Extensor iliotibialis A	Tensor fasciae latae
Extensor iliotibialis B	Gluteus maximus
Cruralis	Cruralis
Puboischiofemoralis internus A	Iliacus internus
Puboischiofemoralis internus B	Iliacus externus
Adductor femoris	Adductor magnus
Pubotibialis A	Sartorius
Pubotibialis B	Semitendinosus
Gracilis major et minor	Gracilis major et minor
Ischioflexorius	Semimembranosus
Caudofemoralis	Pyriformis
Puboischiofemoralis externus A	Pectineus
Puboischiofemoralis externus B	Adductor longus
Ischiotrochantericus B	Obturador externus
Flexor digitorum communis	Plantaris longus
Cruroastralagus	Tibialis posticus
Tarsalis anticus	Tarsalis anticus
Contrahentium caput longum	Tarsalis posticus
Tibialis posterior	Plantaris profundus
Flexores breves superficiales	Flexor digitorum brevis superficialis
Abductor digiti minimi	Abductor brevis dorsalis digiti V
Extensor digitorum longus	Extensor digitorum communis longus
Tibialis anticus	Tibialis anticus
Tibialis anticus brevis	Tibialis anticus brevis
Extensor cruris tibialis	Extensor cruris brevis
Peroneus	Peroneus

**Fig 1:**
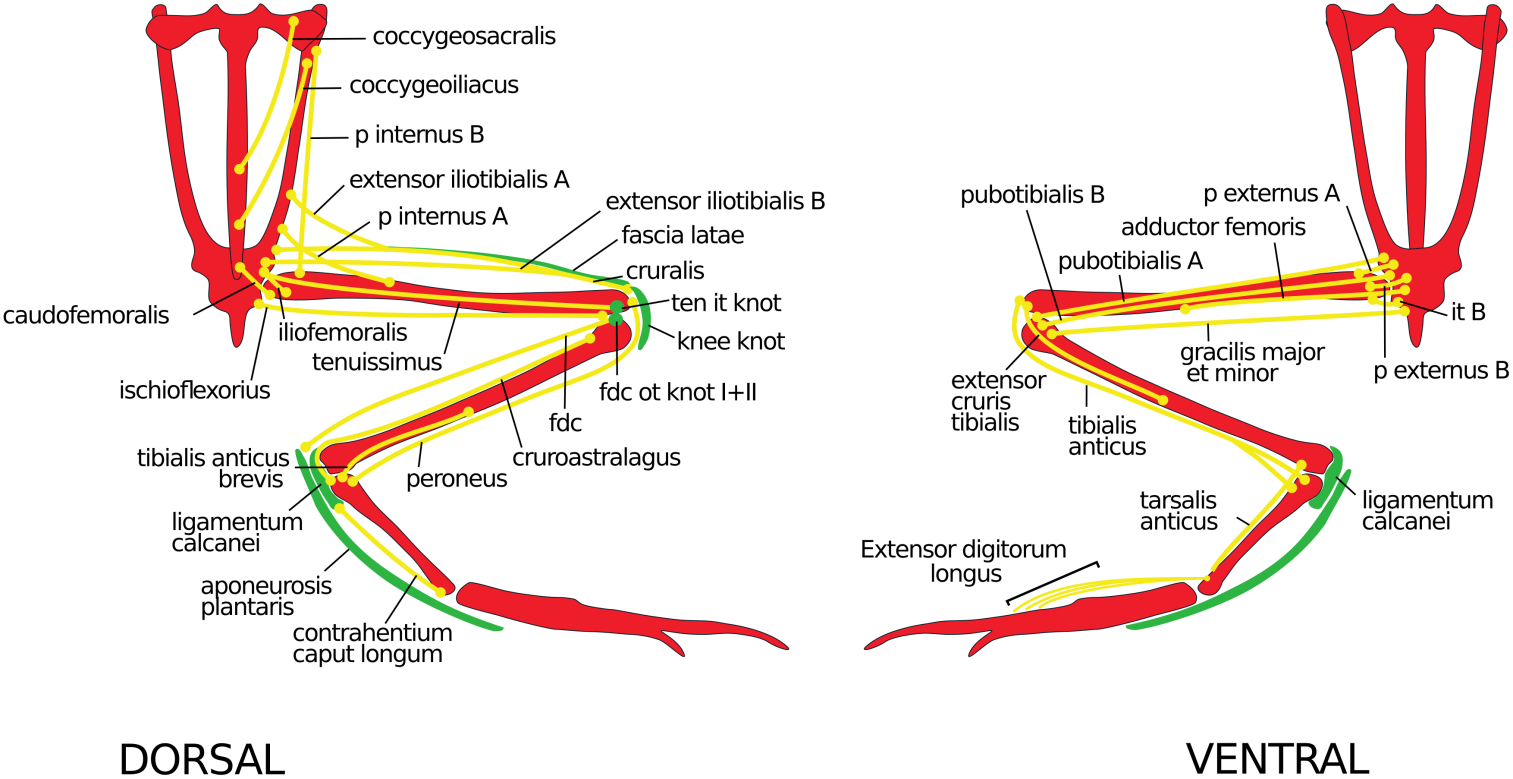
Anatomical elements of the anuran hindlimb considered in this study. Bones in red, muscles in yellow and fibrous knots in green.fdc, flexor digitorum communis; fdcot knot I+II, flexor digitorum communis origin tendon knots I+II; it B, ischiotrochantericus B; p, puboischiofemoralis; ten it knot, tenuissimus insertion tendon knot.

Networks are collections of discrete entities or elements (vertices or nodes) connected by some relationship of interest (links or edges). In our case, nodes correspond to anatomical elements (bones, muscles and fibrous knots) and edges encode physical contacts among such elements (e.g. tendinous and ligamentous junctions). Fig 1 shows the actual anatomical substrate from where the network is constructed.

**Fig 2:**
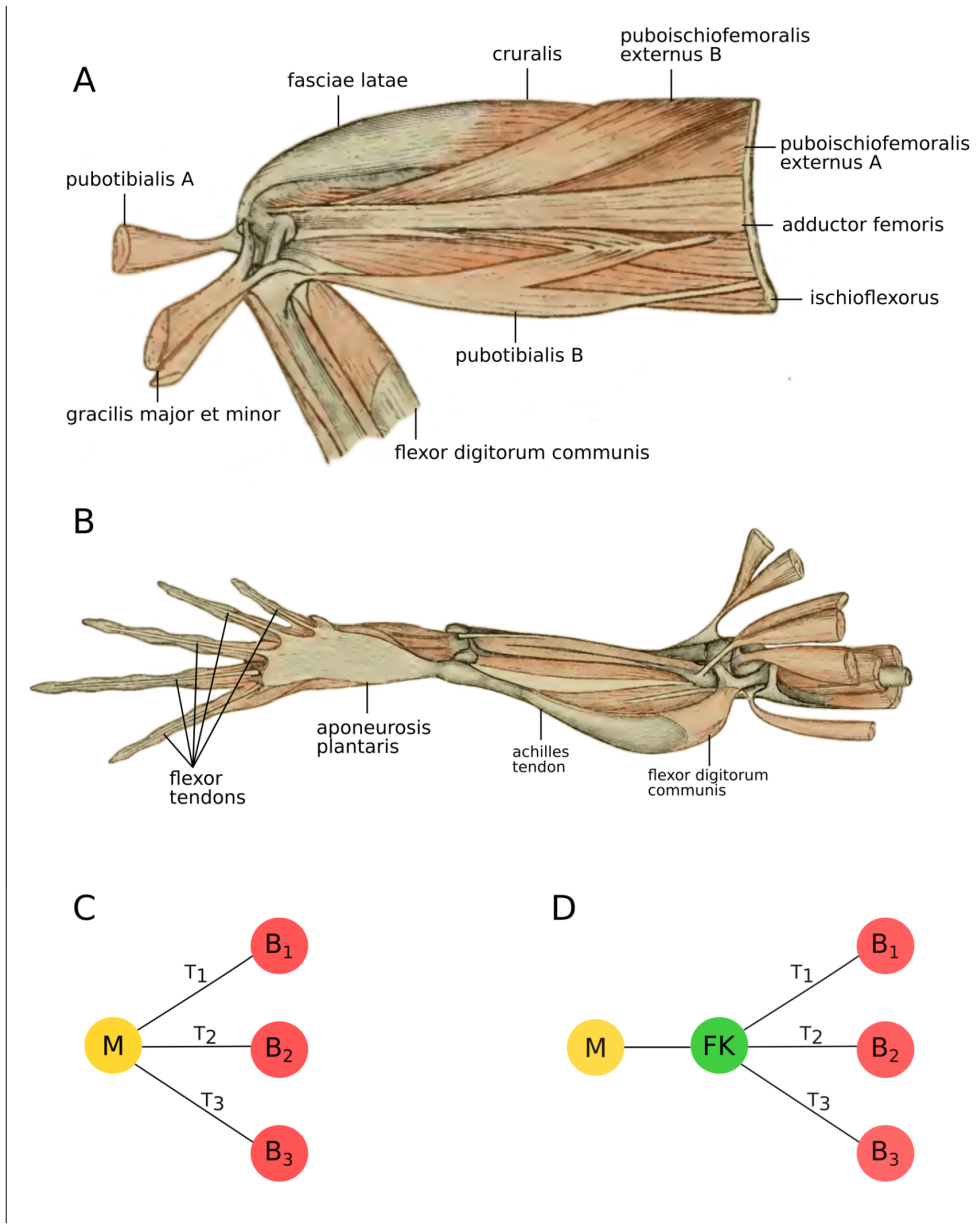
Illustrative example highlighting the concept of connective knot. Anatomical (A and B) representation of the fleshy elements in the hindlimb of a frog. Drawings are modified from the classic work of Gaupp [60]. The Achilles tendon originates from the flexor digitorum communis (fdc) and then spreads out along the underside of the foot to form the aponeurosis plantaris, which in turn sends tendinous strands into each of the several digits. For this particular case, musculoskeletal connections between fdc and digits can be modeled through two alternatives, one more naïve (C) and one more realistic (D) network representation. In the naïve case, several edges are incident on the same muscular node suggesting that several tendons originate from such muscle. In the realistic case, just a single edge is incident on the muscular node, better reflecting the existence of the single Achilles tendon originated from the muscle under consideration. The inclusion of a fibrous knot enables us to better tackle the subtleties in the musculoskeletal connections. B bones; T tendons; M muscles and FK fibrous knots.

Fibrous knots are here defined as critical portions of connective tissues from which multiple (more than two) sets of identically aligned collagen fibers seem to branch out (Fig 2). They are hypothesized to act as gateways in the transmission of mechanical signals. Consequently, edges incident on the fibrous knots represent tendinous segments, or equivalently, they account for fibrous substrates exposed to uniaxial mechanical stress in the actual anatomical system. The adjacency list, with the respective set of neighbors of each node, can be found in Table 2.

**Table 2:** Nodes comprising the musculoskeletal network. Typology and modular classification are reported. The adjacency list column includes the IDs' neighbors of the target vertex.

ID	Node	Type	Adjacencylist	Module
1	Acetabulum	Bone	2, 8, 20, 22, 27, 28, 29, 30, 31, 40, 41, 44, 45, 48	Thigh
2	Adductor femoris	Muscle	1, 22	Thigh
3	Aponeurosis plantaris	connectiveknot	10, 11, 12, 13, 14, 23, 24, 33, 50	Foot
4	Caudofemoralis	Muscle	22, 54	Hip
5	Coccygeoiliacus	Muscle	28, 54	Hip
6	Coccygeosacralis	Muscle	46, 54	Hip
7	Contrahentium caput longum	Muscle	33, 50	Shank
8	Cruralis	Muscle	1, 20, 21, 32	Thigh
9	Cruroastralagus	Muscle	50, 53	Shank
10	Digit I	Bone	3, 34	Foot
11	Digit II	Bone	3, 35	Foot
12	Digit III	Bone	3, 36	Foot
13	Digit IV	Bone	3, 37	Foot
14	Digit V	Bone	3, 38	Foot
15	Distal tarsal 1	Bone	16, 34, 35, 50	Foot
16	Distal tarsal 2–3	Bone	15, 23, 35, 36, 37, 50	Foot
17	Extensor cruris tibialis	Muscle	22, 53	Shank
18	Extensor digitorum longus	Muscle	35, 36, 37, 53	Foot
19	Extensor iliotibialis A	Muscle	21, 28	Hip
20	Extensor iliotibialis B	Muscle	1, 8, 21	Thigh
21	Fascia latae	connectiveknot	8, 19, 20, 32	Thigh
22	Femur	Bone	1, 2, 4, 17, 25, 26, 29, 30, 31, 32, 39, 40, 41, 42, 43, 49, 51	Thigh
23	Fibulare	Bone	3, 16, 37, 38, 39, 50, 51, 53	Foot
24	Flexor digitorum communis	Muscle	3, 25, 26	Calf
25	Flexor digitorum communis ot Knot I	connectiveknot	22, 24, 53	Calf
26	Flexor digitorumcommunis ot Knot II	connectiveknot	22, 24, 53	Calf
27	Gracilis major et minor	Muscle	1, 53	Shank
28	Iliac Shaft	Bone	1, 5, 19, 42, 43, 46	Hip
29	Iliofemoralis	Muscle	1, 22	Thigh
30	Ischioflexorius	Muscle	1, 22	Thigh
31	Ischiotrochantericus B	Muscle	1, 22	Thigh
32	Knee Knot	connectiveknot	8, 21, 22, 49, 53	Thigh
33	Ligamentum calcanei	connectiveknot	3, 7, 53	Shank
34	Metatarsal I	Bone	10, 15	Foot
35	Metatarsal II	Bone	11, 15, 16, 18	Foot
36	Metatarsal III	Bone	12, 16, 18	Foot
37	Metatarsal IV	Bone	13, 16, 18, 23	Foot
38	Metatarsal V	Bone	14, 23	Foot
39	Peroneus	Muscle	22, 23	Foot
40	Puboischiofemoralis externus A	Muscle	1, 22	Thigh
41	Puboischiofemoralis externus B	Muscle	1, 22	Thigh
42	Puboischiofemoralis internus A	Muscle	22, 28	Hip
43	Puboischiofemoralis internus B	Muscle	22, 28	Hip
44	Pubotibialis A	Muscle	1, 53	Shank
45	Pubotibialis B	Muscle	1, 53	Shank
46	Sacral vertebra	Bone	6, 28, 54	Hip
47	Tarsalis anticus	Muscle	50, 53	Shank
48	Tenuissimus	Muscle	1, 49	Thigh
49	Tenuissimus it Knot	connectiveknot	22, 32, 48, 53	Thigh
50	Tibiale	Bone	3, 7, 9, 15, 16, 23, 47, 51, 52, 53	Shank
51	Tibialis anticus	Muscle	22, 23, 50	Shank
52	Tibialis anticus brevis	Muscle	50, 53	Shank
53	Tibiofibula	Bone	9, 17, 18, 23, 25, 26, 27, 32, 33, 44, 45, 47, 49, 50, 52	Shank
54	Urostyle	Bone	4, 5, 6, 46	Hip

The anatomical elements of this study include intrinsic components of the pelvic girdle, stylopodium, and zeugopodyum, and extrinsic elements of the autopodium; except for the short muscles of the acetabular joint (quadratus femoris, gemelus, and obturador internus) (Fig 1). Due to the fact that the distal iliac area, ischium and pubis emerge as a single mesenchymal matrix during development [62,63], and are fused into one element in the adult stage, they were merged into the 'acetabulum' element of the matrix. We consider the same point of origin for all the flexor tendons of the foot, because they integrate the same superficial tendinous layer (i.e. the aponeurosis plantaris) despite their double origin (flexor tendons of digits I, II, and part of III arise from the aponeurosis plantaris and those of digits III, IV and V from m. flexor brevis superficialis). This study is performed with specimens that were already housed in herpetological collections; thus, permissions to collect specimens or the following of ethical regulations were not necessary.

### Network analysis

#### General concepts

A network is a collection of nodes (or vertices) and links (or edges) modeling the elements of a system and their relationships, respectively. Mathematically, networks are studied by the graph theory. Below, we introduce certain technical concepts in order to facilitate the lecture of the manuscript by a broad readership.

Links can be **directed** or **undirected** depending on the oriented nature of the relationship, and **weighted** or **unweighted** if the strength of the relationship is valued or not, respectively. A **path** in a graph represents an alternate sequence of vertices and edges from a certain origin to a certain **destination** by traversing edges. The first vertex of the first edge of a path is the origin and the second vertex of the last edge is the destination. Both origin and destination are called the endpoints of the path. The **length** of a path is the number of edges that it uses. Given a graph G, the **distance** d (x, y) between two vertices x and y is the length of the shortest (or geodesic) path from x to y, considering all the possible paths from x to y in G.

#### Betweenness

Centrality measures capture the relevance of the position of the individual nodes in the network. One of these quantitative indices is the **betweenness** score. Betweenness accounts for the frequency of occurrence of a given node in shortest paths between any pair of nodes in the network. It is useful for detecting the bridging role a given node can exhibit in the flow of information across the structure of the network. As nodes with high betweenness are removed, the remaining elements of the network are prone to be disconnected. In the context of muscle-skeletal networks, centrality indicates high exposure of elements to the propagation of force.

#### Modularity and network layout

Modularity refers to densely connected clusters of nodes embedded into a more global network. It quantifies the extent, relative to a null model network, to which vertices are grouped into dense sub-graphs [3]. The critical issue for quantifying it relies on the definition of a modularity matrix **B** = **A**–**P**, in which **A** is the adjacency matrix of the network that defines a graph with *n* vertices and *m* edges, and the matrix **P** contains the probability of two nodes to be connected by an edge under the prescriptions of a random configuration model. In other words, each element of **P** = [*p*_*ij*_] corresponds to the probability of existence of an edge between vertices *i* and *j* in a random network in which the degrees of all vertices are the same as in the input graph. For unimodal networks, the usual null model sets such probability as proportional to the product of degrees *k* (number of actual neighbors) of the nodes involved, namely *p*_*ij*_ = *k*_i_*k*_*j*_/(2*m*). A measure of modularity, called coefficient Q, is devised in [3] and consists of adding the entries of the modularity matrix for pairs of vertices belonging to the same group of a given vertex set partition. Thus, the modularity Q, for a given assignment *g* of vertices to groups or modules, reflects the extent to which edges are formed within modules instead of between modules, relative to a null model. Formally, modularity is defined as

$$Q=\frac{1}{2m}\underset{}{\sum_{i,j}\left(A_{ij}-P_{ij}\right)\delta \left(g_{i},g_{j}\right)}$$

where the right hand factor is the Kronecker delta, which is equal to 1 if nodes *i* and *j* are in the same module and is otherwise 0. The partition, or division of vertices into modules, that maximizes Q is preferred. Since modularity optimization is NP-hard, heuristic procedures have been developed. Among the many available algorithms implemented in the igraph package of R, we achieve the best performance with the method of Brandes et al. [64] coded in a function named *cluster_optimal*. This method uses an Integer Linear Programming formulation to maximize modularity.

Information about the topology of network relationships is encoded in the adjacency matrix, but structural patterns, such as modularity, can be easily revealed through appropriate network drawings. Force-directed algorithms for the placement of nodes within a rectangular frame of simple undirected graphs are noteworthy. Besides aesthetically pleasing, their output frequently reveals aspects about the organization of links, because such algorithms calculate the layout of a graph using only information contained within the structure of the graph itself. The layout method of Fruchterman & Reingold [65] belongs to that category of graph drawing and is inspired in spring forces proper of natural systems. In this method, the final location of a node is dictated by the interplay between repulsive and attractive forces between nodes. Repulsive forces act among all pairs of nodes, while attractive forces act between adjacent nodes. The final output aims to capture as much symmetry as possible and pursues to evenly distribute the vertices in the frame. Since vertices connected by an edge tend to be drawn near each other, modules can be recognized as proximate subsets of connected nodes. When dealing with sparse small graphs (such as ours), this method is highly recommended for cluster detection. Different analyses and graphics were performed through the add-on package igraph, available in R software [66].

## Results

### Overall network layout

**Fig 3:**
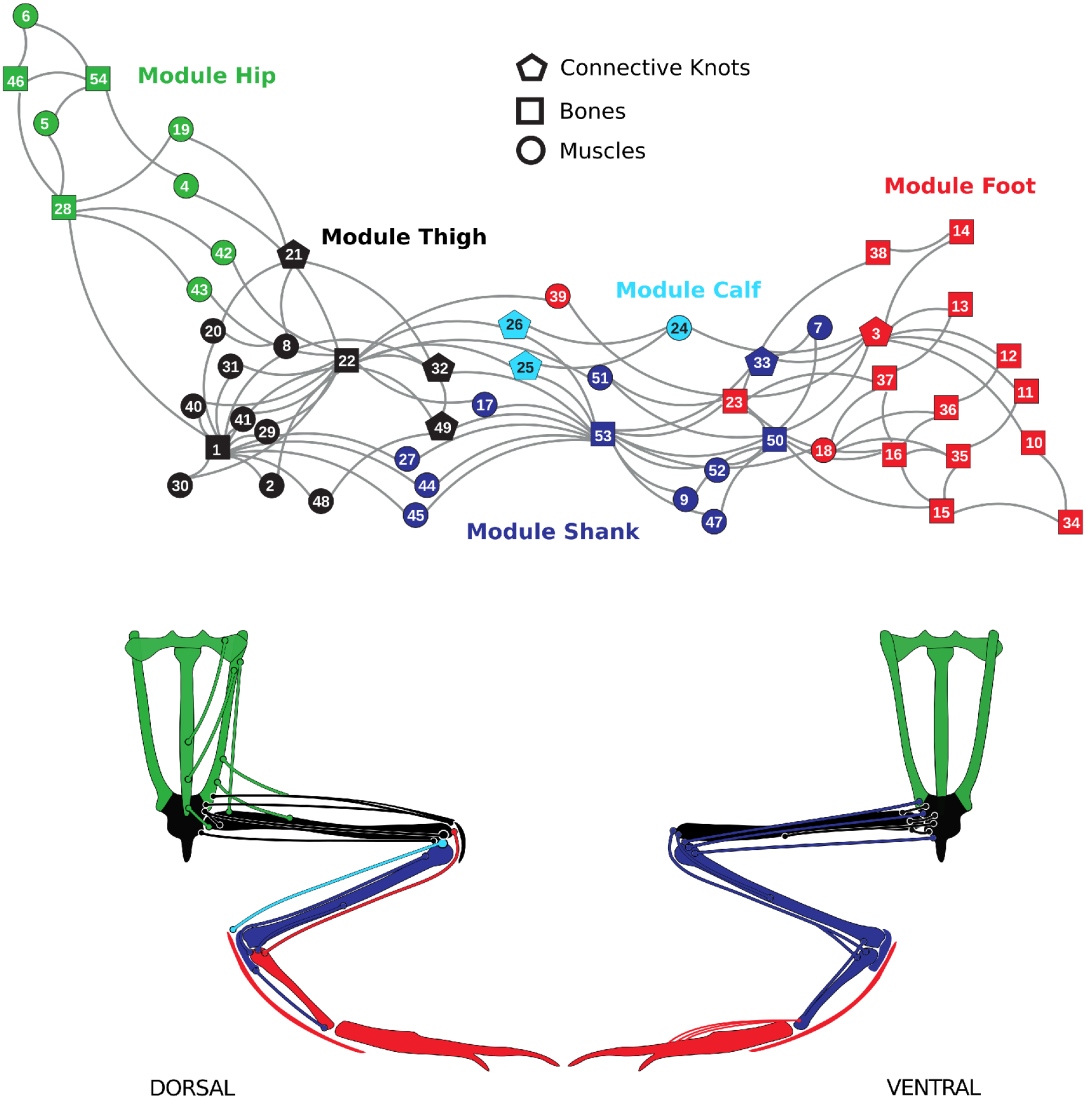
Musculoskeletal network associated with the frog hindlimb. Nodes are colored by common membership to modules. ID numbers are shown in Table 2. The schematic representation of the involved anatomical elements is provided below.

The network associated with the musculoskeletal system of the anuran hindlimb is showed in Fig 3. The two-dimensional arrangement of nodes aims to reflect the modular structure underlying the network. This layout, automatically obtained after applying the Fruchterman-Reinglod algorithm, largely mirrors the overall architecture of the hindlimb: the modular organization and proximal-distal arrangement of elements.

We compiled 27 muscles, 20 bones and 7 fibrous knots as nodes. With the exception of the cruralis and the extensor iliotibialis B, all pairs of muscular elements remain without a direct link between them. Additionally, 22 out of 27 muscles are of degree 2 reflecting origin and insertion into skeletal elements in a simple way.

### Centrality

**Fig 4:**
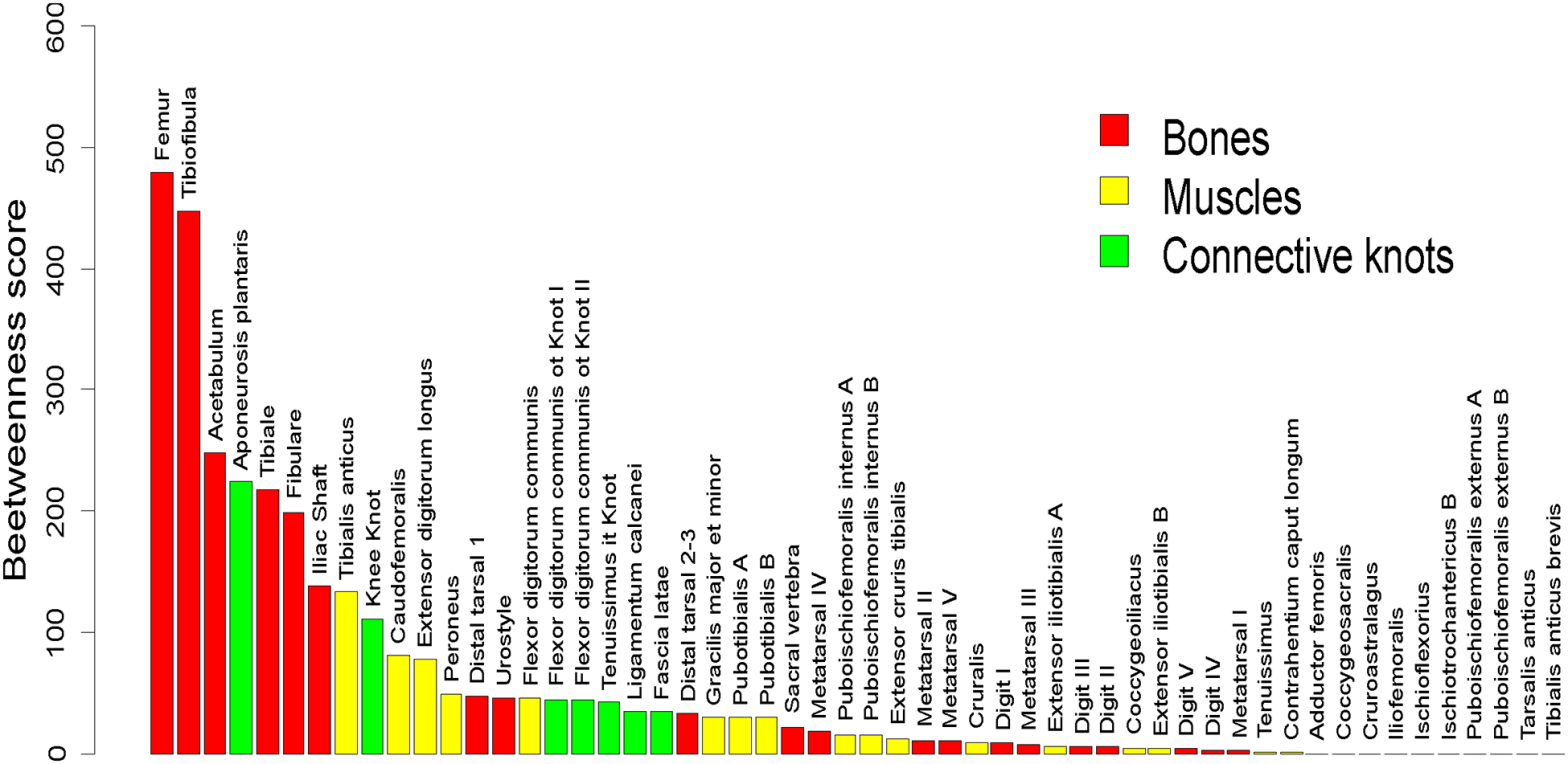
Elements of the anatomical network, ordered by their decreasing betweenness scores.

The system includes a few nodes with higher centrality values. Nine out of 54 nodes (about 17%) show the highest values of betweenness in the data set (larger than 100, Fig 4). The decreasingly ordered sequence of betweenness scores evokes an exponential decay. The osteological elements account for the majority of elements belonging to the set with higher centrality values: femur, tibiofibula, acetabulum, iliac shaft and tibiale. Comparatively, muscles yield the lower centrality values. All the elements related to the autopodium show low betweenness values. A final remark about betweenness profile involves the high performance of fibrous knots such as the aponeurosis plantaris, with a key role in connecting the shank with the feet.

### Modularity

The modules obtained through the usual null model yield a Q value of 0.49 and are represented in Fig 3. Table 3 shows the different elements comprising each module. These modules are interpreted as the following morpho-functional complexes: (1) hip complex, tied with hip movements such as rotation, protraction, and flexion (green nodes in Fig 3); (2) thigh complex, including most of the nodes of the stylopod. This module is related to the distal movements, including adduction, retraction and protraction of the femur and the knee (black nodes in Fig 3); (3) shank complex, embracing the elements associated to the tibiofibula and fibulare, with associated muscles (blue nodes in Fig 3); (4) calf complex, including the prominent flexor digitorum communis and its origin fibrous knots (cian nodes in Fig 3); and (5) foot complex, embracing the fibulare and the multiple bones of the autopodium in addition to one extensor element and to the flexor surface of the foot (red nodes in Fig 3).

**Table 3:** Composition of the modules detected through the network analysis.

Modules	Bones	Fibrous Knots	Muscles	Complex
Module 1 (green)	urostyle, sacral vertebra, iliac shaft	-	coccygeosacralis, coccygeosiliacus, extensor iliotibialis A, caudofemoralis, puboischiofemoralis internus A, puboischiofemoralis internus B	Hip
Module 2 (black)	acetabulum, femur	fascia latae, knee knot, tenuissimus it knot	cruralis, extensor iliotibialis B, tenuissimus, adductor femoris, iliofemoralis, ischioflexorius, ischiotrochantericus B, puboischiofemoralis externus A, puboischiofemoralis externus B	Thigh
Module 3 (blue)	tibiale, tibiofibula	ligamentumcalcanei	Contrahentium caput longum, extensor cruris tibialis, cruroastragalus, gracilis major et minor, pubotibialis A, tarsalis anticus, tibialis anticus, tibialis anticus brevis, pubotibialis B	Shank
Module 4 (cian)	-	flexor digitorumcommunisot knot I, flexor digitorumcommunisotknot II	flexor digitorum communis	Calf
Module 5 (red)	digits I–V, metatarsals I–V, distal tarsals, fibulare	aponeurosis plantaris	extensor digitorum longus, peroneus	Foot

## Discussion

We accept a basic statement of our work in that there is a correspondence between the topographical arrangements of elements and the network topology derived from their physical connections. This finding reinforces the notion that modeling the hindlimb as a network is a valuable resource for testing an old hypothesis from the field of anatomy (e.g. principe des connexions about homologies [67]) and for studying scarcely explored ideas to date, such as tensegrity properties [68].

Bones are among the most central nodes, actually overpassing the fibrous knots. This result contradicts our first hypothesis, which considered the fibrous elements of connective tissue to be more prominent in this regard. Consequently, bones play a leading role on the integrity of the system. The fact that bones exhibit higher centrality values than elements such as fibrous knots is worth remarking, since the latter have been classically considered to present a main role connecting bones and muscles. Our network analysis, however, tells a different story in which bones are the frame connecting muscles and fibrous entities. A similar result was obtained by Diogo et al. [43] in relation to the human hindlimb. These results indicate that the main role of fibrous knots probably resides in their biomechanical properties, which would be much more important than their role as connectors. In fact, most of these fibrous knots tend to undergo metaplasic processes generating fibrocartilage, or even bones (e.g. the fibrocartilaginous patella in the knee knot [69], the plantar sesamoid in the plantar aponeurosis [70], and the cartilaginous sesamoid in the ligamentun calcanei [70]) as a response to the strong mechanical stress to which they are exposed [71]. This is another argument supporting their consideration as particular nodes in our network. Interestingly, in a recent work of Taglaferri et al. [72], the concept of bone-muscle unit is studied once again, without consideration of the tendinous system, thus reinforcing the notion of tendons as biological devices to handle the energetic intake needed to perform body movements, rather than as elements connecting other anatomical structures. Other authors [73,74] also concluded that one of the main functions of tendons is to facilitate joint motion, which exceeds the contractile capacities of the muscles.

There is a certain degree of correlation between osteological elements ordered by betweenness and the ontogenetic chronology of the hindlimbs in tetrapods. This is worth remarking, because it calls for a model of preferential attachment behind the organization of the network [75]. In other words, the osteological nodes which are first to come up are more likely to be connected with other elements that progressively arrive, and are thus prone to capture new connections, increasing their betweenness. In the ontogeny of the anuran hindlimb, the first osteological element to mature (chondrogenesis and osteogenesis) is the femur, followed by the tibiofibula and finally, by the autopodial elements [63]. More precisely, the elements with the higher level of betweenness, femur and tibiofibula, are the first to differentiate in the hindlimb. These elements have a pioneer role in determining a topographical differentiation inside the limb bud [63,76]. Interestingly, the acetabulum exhibits a lower level of betweenness than the long bones of the hindlimb, a rather counter intuitive result when considering the proximal-distal ontogenetic differentiation of the hindlimb. However, Manzano et al. [63] showed that the mesenchimal condensations of the hindlimb long bones originate in their diaphyses when the acetabulum is still in the interzone stage and when no joint cavity is visible, providing evidence of its late differentiation. Indeed, the level of betweenness of the pelvic girdle elements display the same phenomenon: they all exhibit lower level of betweenness than the hindlimb, in a consistent pattern with their ontogenetic sequence. Thus, our data reinforce the idea that the limb is incorporated as an almost complete framework for the development of the pelvic girdle. This topographical arrangement, together with the location of the tendon primordia as described by Manzano et al. [63], likely collaborates in the organization of the position of the muscular complexes [76].

The modularity analysis reveals a clustered organization of the tendon-musculo-skeletal network, supporting the implicit assertion of our second hypothesis. The modular arrangement adjusts to a topographical scheme of proximal-distal organization. Although this conclusion might seem obvious, it should be noted that other results, involving the mixing of distal and proximal elements, could have been obtained. Following this interpretation, the modular arrangement shows evidence of developmental and functional constraints in the structural organization of the frog hindlimb, sustaining thus our third hypothesis. Modules, interpreted as sets of densely connected elements, can be considered as an epiphenomenon comparable to that of anatomical modules from the evo-devo perspective. A clear example of this kind of reciprocal illumination includes the pervasive assignation of the autopodium to a module in its own right [77,78]. The modular construction of vertebrate appendages has been a major factor throughout their evolution. Shubin & Davis [79] conceive modules as existing at several hierarchical levels in limbs, from the whole organ to combinations of their endoskeletal constituents.

Two layers of nodes (skeletal and muscular) seem to exist, in which the elements of the latter are largely unconnected. If the single exception of muscle-to-muscle link is disregarded, the network as a whole would subsume into a type of graph known as quasi-bipartite graph. In a quasi-bipartite graph there are two sets of nodes: one partition with connections among them and another set of nodes with no connection. The adequacy of the quasi-bipartite model for musculoskeletal networks deserves a more in-depth consideration because of its potential impact for modularity studies. Given the explicit dependence of modularity upon the null model, it is clear that the specific choice of the null model largely influences modularity [80]. In fact, a quasi-bipartite graph calls for a different null model than the usual to study modularity, in which the constraints of the absence of a connection for the disjoint set of vertices should be considered. Therefore, the development of a more realistic null model for assessing the modular structure in musculoskeletal networks is of urgent need. For instance, **P** must assign zero likelihood to edges between vertices of the same independent set of vertices, precluding such edges in the null model. Thus, pairs of vertices from the naturally disjoint set should not receive a modularity penalty.

Jumping is a primary locomotion mode in frogs [47,81,82] and particularly in *Leptodactylus latinasus*, an outstanding jumping frog [83]. This remarkable and distinctive function can be disaggregated into functional sublevels, namely rotation, adduction, articulation, extension, etc. The coordinated action performed by modules within the hindlimb is thought to maximize jumping [84]. This is to be expected in the context of evolutionary biology, since modules are considered as autonomous anatomical parts, influenced by local factors and integrated through common developmental factors [40]. Functionally, there is a correlation between the proximal-to-distal succession of modules and the progressive recruitment of elements involved in joint motion during jumping. In a typical jump of a frog, the iliosacral and hip joint movements start almost simultaneously, followed by the knee joint and finally by the ankle joint [84,85]. This sequential movement of joints assures an extended acceleration of the center of mass [84].

The two elements that form the ilium -the iliac shaft and the acetabular portion-, belong to two different modules. One of them denotes the relationship between the pelvic girdle and the body, while the other exhibits a stronger relationship with the proximal elements of the thigh, i.e. the link between the pelvic girdle and the hindlimb. This separation of the same bone in two modules could be interpreted from a functional perspective, since the elongated ilial shafts of anurans are specifically modified for jumping [86]; the acetabular portion, instead, acts as an attachment element with the hindlimbs, through the joint with the head of the femur, in a similar way than in other tetrapods.

The thigh module is mostly related to the flexion and extension of the shank at the knee [87] and is marked by a relatively high percentage of fibrous elements (fascia latae, knee knot, tenuissimus it knot). In the network layout, guided by topological information alone, this module seems to bisect the proximal and distal pools of anatomical elements.

The flexor digitorum communis muscle is clearly the strongest muscle of the hindlimb, and is the main muscle used by frogs in jumping [88]. It is the only muscular element which comprises the calf module, along with its fibrous origin knots. The outer links of this module correspond to the distal part of the femur and the aponeurosis plantaris. This last edge is the Achilles tendon, responsible of the storage of elastic energy for the amplification of power output during jumping [89]. Further studies are needed to assess whether these modules are also recovered in the network of hindlimbs of other species of jumping tetrapods.

The anuran hindlimb presents an additional functional segment composed by the tibiale and fibulare [90,91], which is also incorporated in musculoskeletical modeling [92]. These bones are usually short bones belonging to the autopodium, but in anurans they elongate to form the additional functional segment mentioned above. In our analysis, the tibiale is included in a common module, also integrated by the tibiofibula. Therefore, at least in relation to this bone, our network reinforces the notion of zeugopodization experienced by certain mesopodial bones that receive signals coming from the zeugopodial area to start elongation [93].

The most distal module comprises the many bones of the foot, the aponeurosis plantaris and two muscles which are responsible of the extension of the digits and of the dorsiflexion and pronation of the foot [88]. Diogo et al. [43] included more elements in the autopodial segment than those included in our analysis, and they were able to outline six modules in their study. In our analysis, the elements of the foot are grouped in a single module. Our data show a lower degree of resolution and the discrepancy in the number of modules might be a matter of scale. To conclude, modular architecture seems to be a successful organization that provides the building blocks over which evolution forges the many different functional specializations that organisms can exploit.
